# Expression of Toll-like Receptor 9 in nose, peripheral blood and bone marrow during symptomatic allergic rhinitis

**DOI:** 10.1186/1465-9921-8-17

**Published:** 2007-02-28

**Authors:** Mattias Fransson, Mikael Benson, Jonas S Erjefält, Lennart Jansson, Rolf Uddman, Sven Björnsson, Lars-Olaf Cardell, Mikael Adner

**Affiliations:** 1 Laboratory of Clinical and Experimental Allergy Research, Department of Oto-Rhino-Laryngology, Malmö University Hospital, Lund University, Malmö, Sweden; 2 Department of Pediatrics, Queen Silvia Children's Hospital, Sahlgrenska University Hospital, Gothenburg, Sweden; 3 Department of Experimental Medical Science, Lund University Hospital, Lund University, Sweden; 4 AstraZeneca R&D, Lund, Sweden; 5 Department of Clinical Chemistry, Malmö University Hospital, Lund University, Malmö, Sweden

## Abstract

**Background:**

Allergic rhinitis is an inflammatory disease of the upper airway mucosa that also affects leukocytes in bone marrow and peripheral blood. Toll-like receptor 9 (TLR9) is a receptor for unmethylated CpG dinucleotides found in bacterial and viral DNA. The present study was designed to examine the expression of TLR9 in the nasal mucosa and in leukocytes derived from different cellular compartments during symptomatic allergic rhinitis.

**Methods:**

The study was based on 32 patients with seasonal allergic rhinitis and 18 healthy subjects, serving as controls. Nasal biopsies were obtained before and after allergen challenge. Bone marrow, peripheral blood and nasal lavage fluid were sampled outside and during pollen season. The expression of TLR9 in tissues and cells was analyzed using immunohistochemistry and flow cytometry, respectively.

**Results:**

TLR9 was found in several cell types in the nasal mucosa and in different leukocyte subpopulations derived from bone marrow, peripheral blood and nasal lavage fluid. The leukocyte expression was generally higher in bone marrow than in peripheral blood, and not affected by symptomatic allergic rhinitis.

**Conclusion:**

The widespread expression of TLR9 in the nasal mucosa along with its rich representation in leukocytes in different compartments, demonstrate the possibility for cells involved in allergic airway inflammation to directly interact with bacterial and viral DNA.

## Background

Allergic rhinitis is an inflammatory disorder of the mucosa in the upper airways with infiltration of inflammatory cells like neutrophils, eosinophils, basophils and mast cells [1]. Similar to other atopic diseases, it constitutes a systemic condition where a local allergic reaction may result in distant inflammatory manifestations [2-6]. Bacterial and viral infections are known to worsen allergic rhinitis and induce exacerbations in asthma [7]. Although the pathogenic mechanisms behind this have been extensively investigated, existing data are not conclusive [8]. Toll-like receptors (TLRs) are a group of trans-membrane receptors activated by conserved molecular patterns of microbes [9]. Microbial ligands activate the innate immune system to mount a defense response by binding to TLRs and this process is suggested to be important for an effective presentation of antigens to the adaptive immune system [10]. Consequently, TLRs might be relevant for the pathophysiology of inflammatory airway disorders [11,12]. Ten different TLRs have been described in humans and TLR9 is the receptor for unmethylated CpG dinucleotides, found in bacterial and viral but not in human DNA [13]. Expression of TLR9 has been demonstrated on primary and cultured cells from the human lower airway epithelium and in sinonasal tissue [14,15]. TLR9 has also been found on leukocytes like monocytes/macrophages, B cells and neutrophils as well as in dendritic cells [16,17].

Data regarding the expression of TLRs during periods of airway inflammation is scarce. We have recently demonstrated that an intranasal allergen challenge increased the expression of TLR2, TLR3 and TLR4 in nasal epithelial cells [18]. Patients with vernal keratoconjunctivitis, a chronic allergic inflammation of the ocular surface, have been shown to exhibit reduced mRNA levels of TLR9 in stromal cells [19], but the expression of TLR9 during allergic airway inflammation remains to be explored. Hence, the present study was designed to investigate the expression of TLR9 in human nasal mucosa and in leukocytes derived from bone marrow, peripheral blood and nasal lavage fluid, with focus on compartmental differences and possible changes during symptomatic allergic rhinitis.

## Methods

### Subjects and study design

The study included 32 non-smoking patients (14 women and 18 men) with birch and/or grass pollen induced seasonal allergic rhinitis and 18 non-smoking healthy volunteers (10 women and 8 men), serving as controls. The median (range) age of patients and controls was 27 (18–54) and 26 (22–51) years, respectively. All control subjects were healthy, as were the rhinitis patients with the exception of their allergy.

The expression of TLR9 was assessed in nasal biopsies using immunohistochemistry before and after allergen challenge. Nasal biopsies were obtained from 11 patients at two separate occasions outside pollen season. The first biopsy was obtained during control conditions (outside pollen season and without any prior allergen challenge). 2–4 weeks later, the same patients were challenged intranasally with relevant pollen (birch or grass), and 24 hours after this challenge a second biopsy was obtained from the other nostril. The challenge was performed with 10,000 SQ/U per nostril of Aquagen (ALK, Denmark) with either birch (3 patients) or grass pollen (8 patients). Nine controls were sampled during the same period.

Flow cytometry analysis of TLR9 leukocyte expression was performed on samples obtained during symptomatic allergic rhinitis. Samples of bone marrow, peripheral blood and nasal lavage fluid were obtained from 11 patients with symptomatic allergic rhinitis during either the birch pollen (5 patients) or the grass pollen season (6 patients). They were included at the beginning of the pollen season after having experienced substantial symptoms of rhino-conjunctivitis (itchy nose and eyes, sneezing, nasal secretion and nasal blockage) during at least 3 consecutive days. The majority of patients were seen within 5–10 days after the first appearance of symptoms. A local pollen count confirmed the presence of the relevant types of pollen in the air during this period. In addition, 10 patients with allergic rhinitis and 9 healthy controls were included outside pollen season.

The diagnosis of birch and grass pollen induced allergic rhinitis was based on a positive history of seasonal allergic rhinitis for at least 2 years and a positive skin prick test (SPT) to birch and/or timothy pollen. Patients with seasonal allergic rhinitis had experienced moderate to severe symptoms previous pollen seasons [20,21]. SPT was performed with a standard panel of 10 common airborne allergens (ALK, Copenhagen, Denmark) including pollen (birch, timothy and mugwort), house dust mites (*D. Pteronyssimus *and *D. Farinae*), molds (*Cladosporium *and *Alternaria*) and animal allergens (cat, dog and horse). It was performed on the volar side of the forearm with saline buffer as negative and histamine chloride (10 mg/ml) as positive control. The diameter of the wheal reactions was measured after 20 minutes. All patients presented a wheal reaction diameter >3 mm towards birch or timothy in SPT (roughly corresponding to a 3+ or 4+ reaction when compared to histamine) [22]. Twelve patients presented positive reactions towards both birch and timothy and 8 patients were also positive for mugwort. Patients presenting positive reactions towards animals (8 towards cat, 6 towards dog and 3 towards horse), did not have any regular animal contact. The patients had no symptoms of asthma at the time of visit and they did not take any regular asthma medication (short/long acting β-agonists or inhaled steroids). Exclusion criteria included a history of perennial symptoms, a history of upper airway infection within 2 weeks before the visit and treatment with local or systemic corticosteroids within 2 months before the visit.

The control subjects were symptom-free, had no history of allergic rhinitis and had a negative SPT to the standard panel of allergens described above. They had no history of upper airway infection within 2 weeks before the time of visit and they were all free of medication.

Before inclusion, all subjects, patients as well as controls, were evaluated by an ear-, nose- and throat consultant performing nasoscopy. Individuals with signs or symptoms of chronic rhinosinusitis, hypertrophy of turbinates, severe septum deviation or nasal polyposis were excluded. The study was reviewed and approved by the Ethics Committee of the Medical Faculty, Lund University, and informed consent was obtained from all subjects.

### Symptom and rhinoscopy scores

The subjects were asked to record the severity of three nasal symptoms, i.e. itching/sneezing, secretion and blockage using an arbitrary scale from 0 to 3 (0 = no, 1 = mild, 2 = moderate, 3 = severe symptoms) at the time of inclusion. A total nasal symptom score was calculated by addition of the three scores. Patients challenged with allergen were asked to record a change in this nasal symptom score after 5 and 15 minutes. The maximum of this symptom score was 9. Anterior rhinoscopy was performed on individuals in this part of the study. Oedema and secretion in each nostril were scored from 0 to 2 (0 = no, 1 = mild, 2 = severe). A total rhinoscopy score was calculated by adding the scores for each sign and each nostril. The maximum rhinoscopy score was 8.

### Nasal biopsy procedure

Biopsies were taken from the inferior turbinate after topical application of local anesthesia containing lidocainhydrochloride/nafazoline (34 mg/mL/0.17 mg/mL) for 20 minutes. Biopsies were obtained from 11 allergic patients at two occasions (before and following allergen challenge), and from 9 healthy controls at one occasion.

### Immunohistochemical analysis of TLR9

Nasal biopsies used for immunohistochemistry were frozen in Tissue Tek^® ^O.C.T mounting media (Histo Lab, Gothenburg, Sweden) immediately after excision. Cryosections, 8 μm thick, were after sectioning post-fixed with 2% buffered formaldehyde for 20 minutes, rinsed in phosphate buffered saline (PBS; pH 7.6; 3 × 5 minutes) at room temperature (RT) and placed in 0.1% saponin in PBS for 20 minutes at RT. Non-specific binding sites were blocked with 5% normal serum (DakoCytomation, Glostrup, Denmark; dilution 1:10 in PBS) for 30 minutes. Avidin-binding sites were blocked with incubation of Avidin D solution (Vector Laboratories, Burlingame, CA, USA) for 15 minutes. Thereafter, the sections were rinsed in PBS (3 × 5 minutes) before blocking of biotin-binding sites with biotin blocking solution (Vector Laboratories) for 15 minutes. After additional rinsing (PBS; 3 × 5 minutes) sections were incubated with the primary antibody overnight at 4°C (in control sections the primary antibody was omitted). The primary antibody was diluted in PBS supplemented with 0.25% Triton X and 0.25% bovine serum albumin. The primary antibody, anti-TLR9 (dilution 1:400) was purchased from ImmunoKontact, Oxon, UK. After overnight incubation with primary antibody, the sections were rinsed (3 × 5 minutes in PBS) and incubated with biotinylated secondary antibody (horse anti-mouse IgG1, dilution 1:200, Vector Laboratories) for 45 minutes at RT. After additional rinsing (3 × 5 minutes in PBS), the sections were incubated with alkaline phosphatase-labeled streptavidin (dilution 1:200 for 45 minutes), rinsed (3 × 5 minutes in PBS) and alkaline phosphate activity was developed for 6 minutes at RT using New Fuchsin (DakoCytomation) as enzyme substrate. Endogenous alkaline phosphatase activity was inhibited by Levamisol. No unspecific staining was observed in control sections where the primary antibody was omitted. In additional control experiments, where an isotype-matched antibody was used (M7894, Sigma, Saint Louis, USA), no unspecific staining was found in the nasal epithelium or submucosa. All sections were counter-stained with Harris's hematoxylin, coated with Aqua Perm mounting medium (484975 Life Sci. International), dried overnight and mounted in DPX. Positive immunoreactivity was identified as a bright red precipitate. TLR9 immunoreactivity was assessed and documented by bright field microscopy using an Olympus microscope (Olympus BX) coupled to a high resolution digital camera (Olympus D-50).

### Bone marrow aspiration

One sample containing 1–2 ml of bone marrow was aspirated from the posterior iliac crest following local anesthesia with lidocainhydrochloride (10 mg/ml). The sample was immediately placed in a culture medium containing buffered tri-sodium citrate solution (0.129 M), RPMI 1640 with 2 mM HEPES and N-acetyl-L-alanyl-L-glutamine (FG1233 Biochrom AG, Berlin, Germany). Bone marrow aspiration was obtained from 7 patients with symptomatic allergic rhinitis, from 9 allergic patients outside pollen season and from 8 healthy controls.

### Blood sample collection

One sample containing 4 ml of blood was collected in a test tube containing EDTA (Vacuette^® ^454209) and analyzed for total leukocyte differential count on a cell counter (Beckman Coulter LH750, Marseille, France). An additional sample containing 4 ml of blood was collected in a test tube containing buffered tri-sodium citrate solution (0.129 M, BD Vacutainer™ 367704) and analyzed with flow cytometry. Blood samples were obtained from 11 patients with symptomatic allergic rhinitis, from 10 allergic patients outside pollen season and from 9 healthy controls.

### Recovery of nasal lavage fluid

Nasal lavage fluid was obtained as previously described [23]. Briefly, after clearing excess mucous by forceful exsufflation, 8–10 ml of sterile saline solution (0.9% NaCl) of RT was aerosolized into each nostril, while clearing the other. The nasal fluid was allowed to return passively and collected in a graded test tube, until 7 ml were recovered. The fluids were centrifuged for 10 minutes at 1334 g and 4°C. The pellet, containing the cells, was dissolved in buffered tri-sodium citrate solution (0.129 M) before analysis with flow cytometry. Nasal lavage fluid was obtained from 11 patients with symptomatic allergic rhinitis, from 8 allergic patients outside pollen season and from 8 healthy controls.

### Flow cytometry of leukocytes in bone marrow, peripheral blood and nasal lavage fluid

Bone marrow and nasal lavage samples were filtrated prior to preparation. Analysis was performed for both extracellular (cell membrane) and intracellular occurrence of TLR9. All samples were labeled with CD16-Pcy5 (IM2642, Immunotech, Marseille, France) and CD45-ECD (IM2710, Immunotech) for 15 minutes at RT. For extracellular staining, cells were labeled with TLR9-FITC (211MG3TLR9, ImmunoKontact) for 15 minutes at RT. Erythrocytes in a 50 μl sample were lysed by mixing with 0.6 ml 0.1% (v/v) formic acid for 3–4 seconds. The ionic strength was rendered iso-osmotic by addition of 0.28 ml 51 mM Na_2_CO_3_, 0.20 M Na_2_SO_4 _and 0.22 M NaCl, and cells were washed in PBS and fixed in PBS containing 1% formaldehyde prior to analysis. Intracellular staining was performed using IntraPrep™ Permeabilization Reagent kit (Immunotech) according to the specification of the manufacturer. Thus, the cells were fixed and permeabilized prior to incubation with TLR9-FITC for 15 minutes at RT. Cells were washed in PBS and resuspended in PBS containing 1% formaldehyde prior to analysis. In control experiments (n = 6), cells were also incubated with isotype control antibody, MsIgG_1_-FITC (PN IM0639, Immunotech).

**Figure 1:**
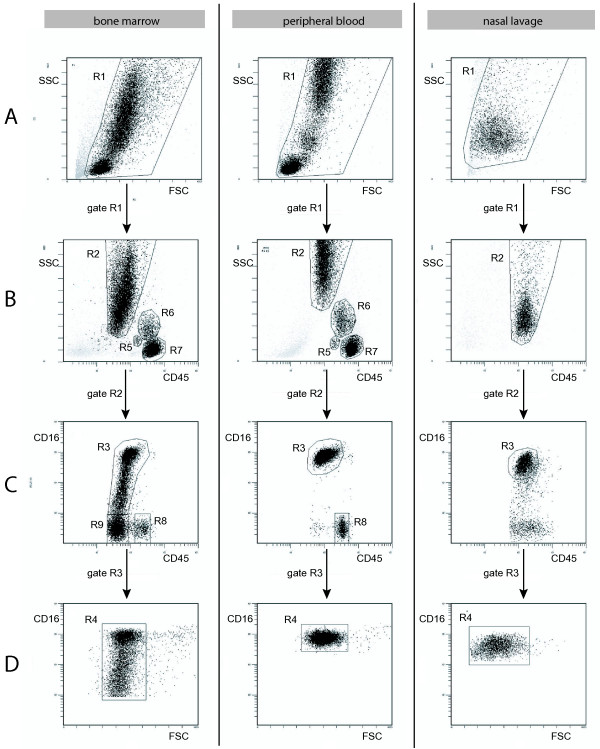
**Leukocyte gates on samples from bone marrow, peripheral blood and nasal lavage fluid**. Flow cytometry data with dot plots showing gates for neutrophils, basophils, monocytes, lymphocytes, eosinophils and immature granulocytes in bone marrow, peripheral blood and nasal lavage fluid. Immature granulocytes were only found in bone marrow. In nasal lavage fluid only neutrophils could be clearly identified. A) FSC versus SSC with gate R1 representing nucleated leukocytes. B) CD45 versus SSC of cells gated from R1, representing basophils (R5), monocytes (R6) and lymphocytes (R7). C) CD45 versus CD16 of cells gated from R2, representing eosinophils (R8) and immature granulocytes (R9). D) FSC versus CD16 of cells gated from R3, representing neutrophils (R4).

By gating intact leukocytes on forward scatter (FSC) and side scatter (SSC) properties as well as by their CD16 and CD45 signals (Figure 1), leukocytes were separated into neutrophils (R4 in Figure 1D), eosinophils (R8 in Figure 1C), basophils (R5 in Figure 1B), monocytes (R6 in Figure 1B) and lymphocytes (R7 in Figure 1B) [24,25]. In addition, immature granulocytes were gated in bone marrow samples (R9 in Figure 1C) [26]. Neutrophil granulocytes were the only cell type that could be clearly identified in nasal lavage fluid. Mean fluorescence intensity ratio (MFIR) was calculated by dividing the mean fluorescence intensity (MFI) for TLR9 antibody with the MFI for the negative control antibody (MsIg) [27,28]. Fluorescence measurement was performed on a Coulter Epics XL flow cytometer (Beckman Coulter). A total of 30,000 events were collected in bone marrow and peripheral blood samples, and 3,000 events were collected in nasal lavage fluid. Data were analyzed using Expo32 ADC analysis software (Beckman Coulter).

An antibody towards a receptor for prostaglandin D2, the chemoattractant receptor homologous molecule expressed on Th2 (CRTH2), known to be highly expressed on peripheral blood eosinophils and basophils [29], was used to assess the purity of eosinophils and basophils. Thus, peripheral blood leukocytes were stained in parallel with CRTH2-PE (PN A07413, Beckman Coulter), CD16-Pcy5 (IM2642, Immunotech) and CD45-ECD (IM2710, Immunotech). Eosinophils and basophils were gated as described above and their CRTH2 signal was examined. In this way, the purity of the eosinophil and basophil gates was determined to 98% and 76%, respectively. The purity of monocytes was determined by staining peripheral blood leukocytes in parallel with CD14-FITC (F0844, DakoCytomation), CD16-PE (R7012, DakoCytomation) and CD45-ECD (IM2710, Immunotech). Monocytes were gated as described above and their CD14 signal was examined. The purity of the monocyte gate was determined to 85%. The purity of neutrophils was determined to 100% with the use of the cell surface marker CD16-Pcy5 (IM2642, Immunotech).

### Statistics

Statistical analysis was performed using the software GraphPad Prism 4 (GraphPad Software, San Diego, USA). All data are expressed as mean ± SEM, and n equals the number of subjects. Kruskal-Wallis test was used in combination with Dunn's Multiple Comparison Test to determine statistical differences. A p-value < 0.05 was considered statistically significant.

## Results

### Symptom and rhinoscopy scores

Patients challenged with allergen reported augmented nasal symptoms. The nasal symptom score increased with 1.3 ± 0.2 (p < 0.001) and 1.2 ± 0.2 (p < 0.001), after 5 and 15 minutes, respectively. Allergic patients examined during pollen season, reported an increase in nasal and eye symptom scores, 4.8 ± 0.6 and 3.9 ± 0.6, compared to allergic patients examined outside season, 0.6 ± 0.3 (p < 0.001) and 0 (p < 0.001), as well as healthy controls, 0.6 ± 0.2 (p < 0.001) and 0 (p < 0.001), respectively. In analogy, the rhinoscopy score in allergic patients was increased during pollen season, 3.0 ± 0.6, in comparison to allergic patients examined outside season, 1.1 ± 0.3 (p < 0.05), and controls, 0.2 ± 0.1 (p < 0.001).

### Immunohistochemical staining of TLR9 in the nose

**Figure 2:**
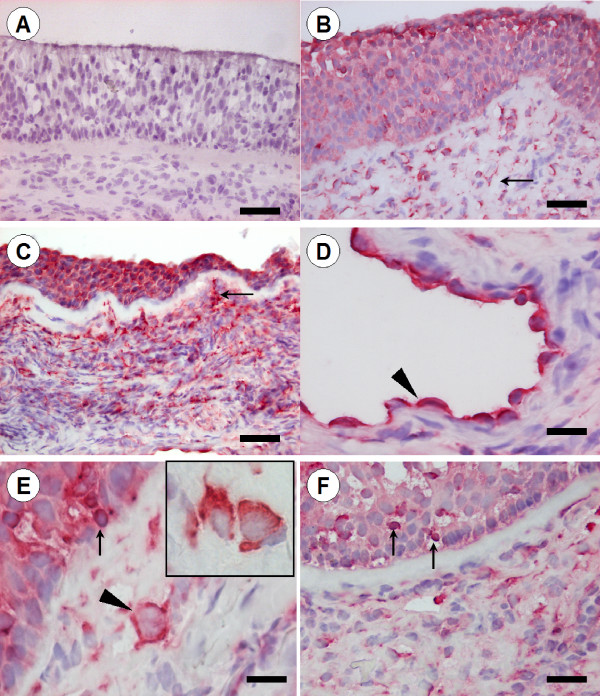
**TLR9 immunoreactivity in the nasal mucosa**. Immunohistochemical localization of TLR9 in biopsies of nasal mucosa is depicted in (B-F) whereas (A) illustrates a representative picture of a control slide. A) No immunoreactivity was observed in control sections where an isotype-matched control antibody was used. B) In an adjacent section, immunoreactivity for TLR9 is seen in the apical part of the epithelial lining, in scattered intra- and subepithelial leukocytes and in elongated fibroblast-like cells in the subepithelial tissue (arrow). The epithelial TLR9 immunoreactivity varied from being foremost present within the apical region of the epithelium (B) to a more even distribution (C). D) A distinct TLR9 immunoreactivity was also present in endothelial cells (arrowhead). E) Bright field micrographs demonstrating TLR9-positive large non-granulated mononuclear cells (arrowhead) and mast cells (inset). F) TLR9-positive intraepithelial lymphocytes (arrows E-F). Scale bars: A-C = 50 μm, D-E = 20 μm, and F = 350 μm.

Immunoreactivity for TLR9 was seen in many different cell types within the epithelium and submucosa of the nose (Figure 2). The distribution pattern of the epithelial staining differed between subjects, in some subjects the staining was foremost distributed to epithelial cells positioned in the apical region of the epithelium (Figure 2B), whereas in others, the staining was equally distributed in the whole epithelial layer (Figure 2C). Overall, the distribution was similar between healthy controls and allergic patients, and it was not changed by the allergen challenge. A distinct TLR9 immunoreactivity was also found in the endothelial cells lining small venules and capillaries (Figure 2D) and in subepithelial structural cells, tentatively identified as fibroblasts (Figure 2C). Immunoreactivity for TLR9 was also seen in scattered intraepithelial and subepithelial leukocytes (Figure 2C). The identification of these cells was based on morphological criteria and in this regard, mast cells were identified as large granulated mononuclear cells, macrophages and dendritic cells as large agranular mononuclear cells, granulocytes by their characteristic polymorph nuclei and lymphocytes as small mononuclear cells with a circular nucleus surrounded by only a thin rim of cytoplasm. Using these morphological criteria, TLR9 immunoreactivity was identified in mast cells (inset Figure 2E), dendritic cells (Figure 2E), granulocytes and lymphocytes (Figure 2E–F). There was no difference in the expression of leukocyte-associated TLR9 between healthy controls and allergic patients, and an altered expression could not be detected after the allergen challenge.

### Total leukocyte counts and cell distributions in peripheral blood and bone marrow

Total leukocyte counts in peripheral blood were similar among the three groups, 6.0 ± 0.4 × 10^6 ^cells/ml in controls, 5.3 ± 0.4 × 10^6 ^cells/ml in allergic patients outside pollen season and 6.5 ± 0.4 × 10^6 ^cells/ml in allergic patients during season. The proportion of neutrophils, eosinophils, basophils, monocytes, and lymphocytes in peripheral blood and bone marrow, and the percentage of immature granulocytes in bone marrow did not differ between the three groups (data not shown).

### Leukocyte expression of TLR9 in bone marrow, peripheral blood and nasal lavage fluid

**Figure 3:**
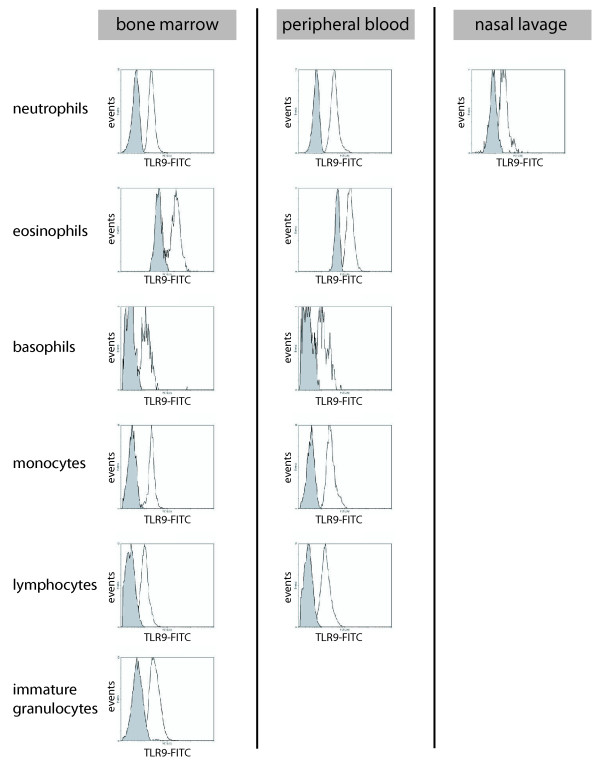
**Expression of TLR9 in leukocytes from bone marrow, peripheral blood and nasal lavage fluid**. Histogram plots of intracellular staining of TLR9 in neutrophils, eosinophils, basophils, monocytes, lymphocytes and immature granulocytes. Expression of TLR9 in leukocytes was analyzed by flow cytometry using mAbs against human TLR9 (open histograms). Cells were fixed and permeabilized prior to incubation with mAbs. Shaded histograms represent cells labeled with isotype-matched control Ab. The data shown were obtained from a control subject and they are representative of those from six independent experiments.

In bone marrow, an intracellular expression of TLR9 was found in neutrophils, eosinophils, basophils, monocytes, lymphocytes and immature granulocytes (Figure 3). No extracellular expression was found on bone marrow leukocytes. In peripheral blood, a similar intracellular expression of TLR9 was found in neutrophils, eosinophils, basophils, monocytes and lymphocytes (Figure 3). A low extracellular expression was found on monocytes (data not shown). Neutrophils were the only cell type that could be clearly identified by flow cytometry analysis in nasal lavage fluid. The number of cells found in nasal lavage fluid varied considerably between individuals, and generally fluids sampled during pollen season yielded the highest cell content. Intracellular expression of TLR9 was evident in neutrophils in nasal lavage fluid (Figure 3).

### Mean fluorescence intensity ratio of TLR9 in different compartments and cell types

**Figure 4:**
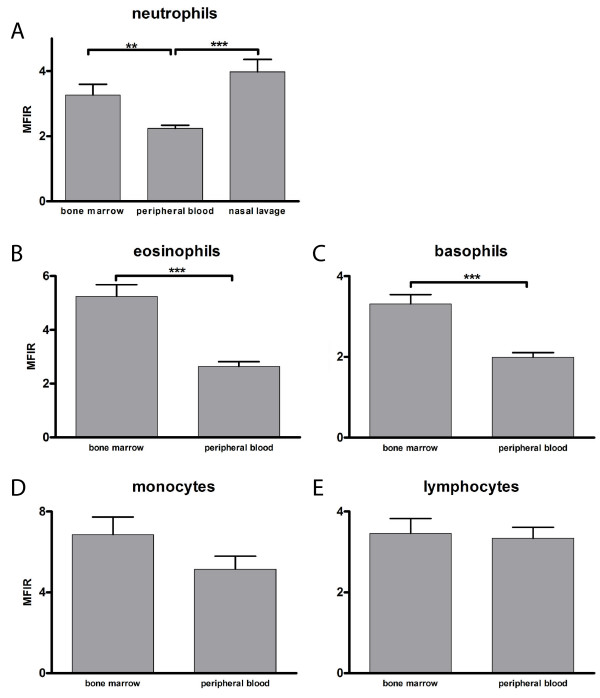
**Expression of TLR9 in leukocytes in different compartments**. Intracellular expression of TLR9, presented as MFIR, in bone marrow, peripheral blood and nasal lavage fluid. Expression of TLR9 in A) neutrophils (n = 23–28), B) eosinophils (n = 23–29), C) basophils (n = 23–27), D) monocytes (n = 23–29) and E) lymphocytes (n = 23–29). Data are presented as mean ± SEM. ** p < 0.01, *** p < 0.001

First, the intracellular expression of TLR9, as measured by MFIR, was compared between the different compartments irrespective of the atopic status of the individuals from which the cells were obtained. The intracellular expression of TLR9 in neutrophils was found to be higher in bone marrow and nasal lavage fluid, 3.26 ± 0.33 and 3.98 ± 0.38, respectively, compared to in peripheral blood, 2.24 ± 0.10 (p < 0.001 and p < 0.01, respectively; Figure 4A). The expression in eosinophils and basophils was higher in bone marrow, 5.24 ± 0.43 and 3.31 ± 0.23, compared to in peripheral blood, 2.64 ± 0.18 and 1.99 ± 0.12, respectively (p < 0.001, Figure 4B–C). There was no difference in the expression of TLR9 in monocytes and lymphocytes in bone marrow, 6.85 ± 0.88 and 3.46 ± 0.36, compared to peripheral blood, 5.14 ± 0.65 and 3.34 ± 0.27, respectively (Figure 4D–E).

**Figure 5:**
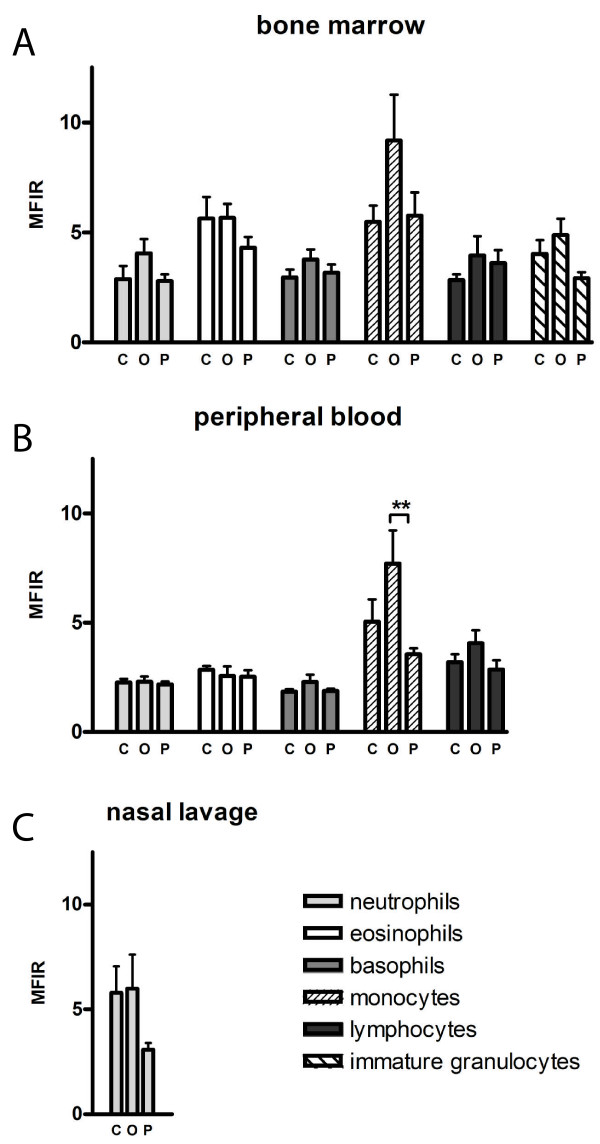
**Expression of TLR9 in leukocytes during allergic rhinitis**. Intracellular expression of TLR9, presented as MFIR, in different leukocytes in healthy controls (C), allergic patients outside season (O) and allergic patients during pollen season (P). Expression of TLR9 in neutrophils, eosinophils, basophils, monocytes, lymphocytes and immature granulocytes analyzed by flow cytometry. Expression of TLR9 in leukocytes in A) bone marrow (n = 23), B) peripheral blood (n = 27–29) and C) nasal lavage fluid (n = 27). Data are presented as mean ± SEM. ** p < 0.01

Next, the influence of allergic inflammation on the leukocyte expression of TLR9 was examined. The levels of intracellular TLR9 expression, as determined by MFIR, were compared between healthy controls, allergic patients outside pollen season and patients during season in each cell type (Figure 5A–C). The expression of TLR9 in peripheral blood monocytes was lower in patients during pollen season, 3.56 ± 0.27, compared to patients outside season, 7.70 ± 1.53 (p < 0.01, Figure 5B).

## Discussion

A distinct expression of TLR9 was found in the epithelium, in inflammatory cells in the submucosa, in the endothelial lining and in structural cells in the nose. TLR9 expression could also be demonstrated in permeabilized neutrophils, eosinophils, basophils, monocytes, lymphocytes and immature granulocytes derived from bone marrow, peripheral blood and nasal lavage fluid. Neutrophils, eosinophils and basophils had a higher expression of TLR9 in bone marrow than in peripheral blood. The onset of symptomatic allergic rhinitis did not affect the TLR9 expression in any of the compartments investigated.

mRNA expression of TLR9 has been demonstrated in sinonasal tissue and expression of TLR9 mRNA and protein has been reported in human cell lines and primary cells of lower airway epithelium [14,15]. Expression of functional TLR9 was detected in a study using a human bronchial epithelial cell line [30]. In the present study, expression of TLR9 was found in the endothelial lining of small blood vessels. This finding is in line with the detection of TLR9 mRNA in mouse lung endothelial cells [31]. Structural cells, proposed to be fibroblasts, showed a variable expression of TLR9 in accordance with a previous study [19]. Expression of TLR9 was found in different inflammatory cells (i.e. leukocytes) in the epithelial and subepithelial region. Based on morphological data, some of these cells were identified as mast cells and dendritic cells. Whereas expression of TLR9 has been demonstrated in human peripheral blood-derived cultured mast cells [32,33], mast cells in the conjunctiva did not stain for TLR9 protein [19]. It is possible that the expression of TLR9 in mast cells is variable in different localizations and among different subtypes of mast cells. Immunohistochemical analysis suggested an expression of TLR9 in intra- and subepithelial lymphocytes and granulocytes.

In accordance with previous studies, we found a significant intracellular expression of TLR9 in neutrophils and eosinophils using flow cytometry [17,19,34]. In addition, TLR9 was expressed in basophils, monocytes and lymphocytes. Only monocytes exhibited a low cell surface expression of TLR9. This finding is in agreement with a previous study where a minor proportion of freshly isolated CD14^+ ^monocytes and B-cells were shown to express TLR9 on their cell surfaces [16]. Human tonsil B cells express high amounts of TLR9 mRNA and protein as determined by Western blot [35], which together with a low surface staining indicate a predominantly intracellular expression of TLR9 in lymphocytes. Unstimulated TLR9 is retained in the endoplasmatic reticulum [36] and upon stimulation, it colocalizes with CpG oligodeoxynucleotide (ODN) in a vesicular lysosomal compartment where signaling is initiated [37]. Following CpG stimulation, a minor portion of TLR9 becomes surface accessible [37], and this might account for the low surface staining found on monocytes in the present study [16]. Intracellular expression of TLR9 was found in bone marrow leukocytes. Expression of TLR9 mRNA has been reported in bone marrow-derived mast cells, plasmacytoid and myeoloid DCs [38], but to our knowledge, there are no previous studies that have demonstrated the expression of TLR9 in human bone marrow leukocytes. In nasal lavage fluid, neutrophils displayed an intracellular expression of TLR9.

The expression of TLR9 in neutrophils, eosinophils and basophils was higher in bone marrow compared to peripheral blood. Such a difference was not seen in monocytes and lymphocytes. Thus, it is possible that the expression of TLR9 has a role in the development and differentiation of granulocytes. The high expression of TLR9 in immature (CD16-negative) granulocytes in bone marrow supports this view. It is also possible that the bone marrow functions as a reservoir of granulocytes with an increased expression of TLR9, ready to be discharged in case of an infection. The nasal epithelium is constantly exposed to the external environment with microbial components and particles. This gives rise to a state of "physiological inflammation" associated with a continuous recruitment of neutrophils to the surface of the nasal mucosa [39]. The increased expression of TLR9 in nasal lavage fluid indicates either that neutrophils expressing high levels of TLR9 are preferentially recruited to the nose (via the bloodstream from the bone marrow reservoir), or that neutrophils are primed with an increased expression of TLR9 as they migrate towards the nasal lumen.

Previously, we reported an increased protein expression of TLR2, TLR3 and TLR4 in the nasal epithelium following allergen challenge [18]. In another study, expression of TLR4 was upregulated and TLR9 downregulated in subjects with vernal keratoconjunctivitis compared to healthy controls [19]. In contrast to this, expression of TLR9 in the nasal tissue was not affected by allergen challenge. In analogy, we did not detect any differences in the leukocyte expression of TLR9 during allergic airway inflammation in bone marrow, peripheral blood or nasal lavage fluid. The relevance of the decrease seen in the expression of TLR9 in peripheral blood monocytes during pollen season is uncertain, since this was not accompanied by a significant decrease in the bone marrow. Even though we did not find any differential expression of TLR9 in patients with symptomatic allergic rhinitis, this does not exclude the possibility that cells could influence the subsequent allergic inflammation through TLR9. In murine models of asthma and allergy, TLR9 stimulation with CpG during as well as after the sensitization procedure, appears to decrease the inflammatory process [40]. In analogy, CpG has been reported to function as an adjuvant for immunotherapy in humans [41]. Thus, the distinct and general expression of TLR9 in the nasal epithelium and in various leukocytes in the nasal mucosa, indicates that bacterial and viral airway infections might affect the adaptive immune response directly by their content of CpG and this could represent one pathogenic mechanism explaining the worsening of allergic inflammation during airway infections.

## Conclusion

The widespread and rich expression of TLR9 in nasal epithelial cells and on nearly all types of leukocytes derived from bone marrow, peripheral blood and nasal lavage fluid, indicates a broad opportunity for bacterial and viral airway infections to interact directly with the adaptive immune response via expression of CpG-related ligands.

## Competing interests

The study was financially supported by the Royal Physiographic Society in Lund, the Swedish Medical Research Council, the Swedish Heart-Lung Foundation, the Swedish Association for Allergology and the Tore Nilsson Foundation of Medical Research.

Lennart Jansson is employed by AstraZeneca R&D.

## Authors' contributions

MF acquired and analyzed the flow cytometry data and drafted the manuscript. JE and RU acquired and analyzed the immunohistochemistry data and revised the content of the manuscript. SB contributed to the analysis of the flow cytometry data and revised the data of the manuscript. MB and LJ contributed to the study-design and revised the data of the manuscript. LOC conceived and coordinated the study. MA participated in the design and helped to draft the manuscript.
